# Deep learning‐based detection and classification of multi‐leaf collimator modeling errors in volumetric modulated radiation therapy

**DOI:** 10.1002/acm2.14136

**Published:** 2023-08-26

**Authors:** Sae Nakamura, Madoka Sakai, Natsuki Ishizaka, Kazuki Mayumi, Tomotaka Kinoshita, Shinya Akamatsu, Takayuki Nishikata, Shunpei Tanabe, Hisashi Nakano, Satoshi Tanabe, Takeshi Takizawa, Takumi Yamada, Hironori Sakai, Motoki Kaidu, Ryuta Sasamoto, Hiroyuki Ishikawa, Satoru Utsunomiya

**Affiliations:** ^1^ Department of Radiation Oncology Niigata Neurosurgical Hospital, Nishi‐ku Niigata City Niigata Japan; ^2^ Department of Radiology Nagaoka Chuo General Hospital, Nagaoka Nagaoka Niigata Japan; ^3^ Department of Radiology and Radiation Oncology Niigata University Graduate School of Medical and Dental Sciences, Chuo‐ku Niigata City Niigata Japan; ^4^ Department of Radiology Niigata Prefectural Shibata Hospital Shibata City Niigata Japan; ^5^ Department of Radiological Technology Niigata University Graduate School of Health Sciences, Chuo‐ku Niigata City Niigata Japan; ^6^ Department of Radiology Takeda General Hospital, Aizuwakamatu City Fukushima Japan; ^7^ Division of Radiology Nagaoka Red Cross Hospital Nagaoka City Niigata Japan; ^8^ Department of Radiation Oncology Niigata University Medical and Dental Hospital, Chuo‐ku Niigata City Niigata Japan; ^9^ Section of Radiology Department of Clinical Support Niigata University Medical and Dental Hospital, Chuo‐ku Niigata City Niigata Japan

**Keywords:** deep learning, multileaf collimator, volumetric modulated arc therapy

## Abstract

**Purpose:**

The purpose of this study was to create and evaluate deep learning‐based models to detect and classify errors of multi‐leaf collimator (MLC) modeling parameters in volumetric modulated radiation therapy (VMAT), namely the transmission factor (TF) and the dosimetric leaf gap (DLG).

**Methods:**

A total of 33 clinical VMAT plans for prostate and head‐and‐neck cancer were used, assuming a cylindrical and homogeneous phantom, and error plans were created by altering the original value of the TF and the DLG by ± 10, 20, and 30% in the treatment planning system (TPS). The Gaussian filters of σ=0.5 and 1.0 were applied to the planar dose maps of the error‐free plan to mimic the measurement dose map, and thus dose difference maps between the error‐free and error plans were obtained. We evaluated 3 deep learning‐based models, created to perform the following detections/classifications: (1) error‐free versus TF error, (2) error‐free versus DLG error, and (3) TF versus DLG error. Models to classify the sign of the errors were also created and evaluated. A gamma analysis was performed for comparison.

**Results:**

The detection and classification of TF and DLG error were feasible for σ=0.5; however, a considerable reduction of accuracy was observed for σ=1.0 depending on the magnitude of error and treatment site. The sign of errors was detectable by the specifically trained models for σ=0.5 and 1.0. The gamma analysis could not detect errors.

**Conclusions:**

We demonstrated that the deep learning‐based models could feasibly detect and classify TF and DLG errors in VMAT dose distributions, depending on the magnitude of the error, treatment site, and the degree of mimicked measurement doses.

## 1 INTRODUCTION

Volumetric modulated arc therapy (VMAT) is widely used for cancer radiation therapy, and multi‐leaf collimators (MLCs) are an essential ingredient in the delivery. The accurate modeling of an MLC in order to specify its characteristics as part of the commissioning of a treatment planning system (TPS) is undoubtedly important for ensuring agreement between dose calculation and measurement.^1^ For example, the Eclipse TPS (Varian Medical Systems, Palo Alto, CA) provides two adjustable parameters for MLC modeling: the transmission factor (TF) and the dosimetric leaf gap (DLG).

Establishing a standard methodology to optimize MLC modeling parameters has been challenging. Some institutional surveys have revealed that MLC modeling parameters showed non‐negligible variations, even among otherwise identical linacs and TPSs.^2^, ^3^ Such variations can be attributed to differences in dose measurement tools and/or inter‐institutional differences of methods to determine the parameters.^2^, ^3^, ^4^, ^5^ It implies that some institutions possibly had failed to adjust the MLC modeling parameters and dose calculation accuracy has been suboptimal. Recently, there have been several proposals by which specific test fields and/or VMAT fields were used to optimize the parameters based on gamma passing rates.^6^, ^7^, ^8^ Xue et al. provided a simple method to obtain the optimal DLG value using clinical VMAT plans and measured dose distribution with an ArcCHECK diode array (Sun Nuclear).^7^ DiCostanzo et al. used two standard intensity modulated radiation therapy (IMRT) commissioning plans and measured doses with a MapCHECK2 diode array (Sun Nuclear) to optimize the DLG and TF based on the mean gamma‐index as the cost function during optimization.^8^ Those studies relied on the method of gamma analysis, which is the most accepted method of evaluating the degree of agreement between calculated and measured dose distributions in 2D and 3D.^9^, ^10^ However, it has been demonstrated that the comparison between measured and TPS‐calculated dose distribution using gamma analysis fails to detect MLC modeling errors.^11^, ^12^, ^13^, ^14^ The insensitivity of the conventional method to MLC modeling error may be due to the smallness of the error and/or the use of gamma analysis. A novel method that can be easily implemented and sufficiently sensitive to small error may be required.

Machine learning and deep learning‐based models have been increasingly used in radiation therapy quality assurance (QA).^15^, ^16^, ^17^ There have been several applications of machine learning or deep learning to patient‐specific QA of IMRT/VMAT aiming to detect and/or classify several types of error which are not just relying on the gamma analysis.^18^, ^19^, ^20^, ^21^, ^22^, ^23^, ^24^, ^25^ Potter et al. adopted an artificial neural network (ANN) and a convolutional neural network (CNN) to detect and classify the six classes of dosimetric errors including the incorrect TF.^21^ Our previous study proposed machine learning models to distinguish MLC modeling errors from MLC positional error using radiomic features of fluence maps measured in patient‐specific IMRT QA with an electric portal imaging device (EPID).^24^ Although this work was successful in detecting TF and DLG errors as distinct from MLC positional errors, the accuracy of discriminating between the TF and DLG errors were reduced to be about 0.6. Previous works left various tasks for the future, including a determination of the feasibility conditions for discriminating between TF and the DLG error and quantitative estimation of the MLC modeling errors. Furthermore, since MLC modeling errors are not as noticeable in dose distributions compared to MLC positional errors, the detectability is likely reduced by measurement noise. Therefore, it is also meaningful to investigate the detectability condition of the MLC modeling errors by simulating a variable measured dose.

In this study, we focused on the detection and the classification of MLC modeling errors by deep learning‐based models. We adopted a detector‐independent method in which a measured dose map was simulated by applying multiple Gaussian filters to search for feasibility conditions of detection and classification between TF and the DLG errors. The proposed models may be useful in performing accurate and efficient MLC modeling in TPS commissioning for VMAT.

## 2 MATERIALS AND METHODS

### 2.1 Simulation of MLC modeling errors

We used 33 clinical VMAT plans from patients at our institute between October 2019 and December 2020 delivered with 6X beams of the Novalis Tx (Varian Medical Systems, Palo Alto, CA). This retrospective study was approved by the institutional review board of our institute. We analyzed 20 prostate cancer plans with 20 arcs and 13 head‐and‐neck cancer plans with 20 arcs, generated using the Eclipse TPS (ver. 15.5, Varian Medical Systems, Palo Alto, CA) with a dose calculation grid size of 2.0 mm. We recalculated dose distributions in a cylindrical and homogeneous numerical Delta4 phantom (22 cm diameter, 40 cm length; ScandiDos, Uppsala, Sweden) for each arc of the clinical VMAT plans using the anisotropic analytical algorithm (AAA) ver. 15.5. We intentionally created TF and DLG error plans by altering the original TF (1.22%) and DLG values (0.88 mm) by ± 10, 20, and 30% relative to the original Eclipse value and then recalculating the dose distributions. We then obtained three types of treatment plan: (a) error‐free plans in which the original values of the TF and the DLG were used, (b) plans with TF error, and (c) plans with DLG error. Three‐axial 2D dose distributions (at isocenter, 1 cm superior and inferior away from the isocenter), three‐coronal 2D dose distribution (at isocenter, 1.5 cm anterior and posterior away from the isocenter), and three‐sagittal 2D dose distribution (at isocenter, 1.5 or 2.0 cm left, and 1.5 cm right) were extracted; hence, 9 dose distributions were obtained for each arc of the clinical VMAT plan.

**FIGURE 1:**
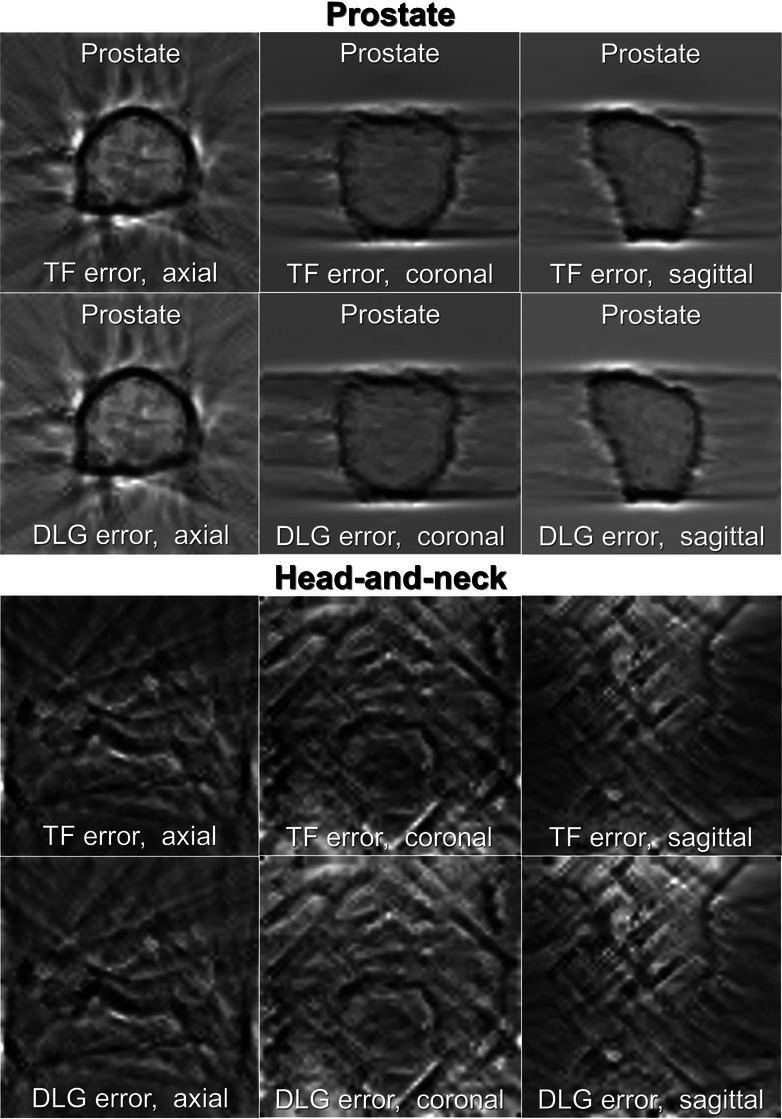
The axial, coronal, and sagittal dose difference map for the +30% TF error in a prostate plan (1st row), the +30% DLG error in a prostate plan (2nd row), the +30% TF error in a head‐and‐neck plan (3rd row), and the +30% DLG error in a head‐and‐neck plan (4th row), respectively. DLG, dosimetric leaf gap; TF, transmission factor.

To investigate the feasibility conditions for detecting MLC modeling errors in measurement, we created multiple patterns of mimicked measurement dose maps by applying a Gaussian filter of two standard deviations (*σ* = 0.5, and 1.0) for the error plans to reproduce a simulated measured dose map similar to those in the method adopted by Potter et al.^21^ We then applied a median filter to all the obtained dose distributions. We created dose difference maps by subtracting calculated dose maps with each error including error‐free maps from the mimicked measured dose maps. The obtained dose difference maps were resampled by linear interpolation to a pixel size of 256 × 256 (one pixel corresponds to 0.391 mm) and converted to an 8‐bit PNG‐file format. The dose difference maps were used for both deep learning modeling and gamma analysis. Finally, the total number of dose difference maps of error‐free plans was 360 while that of the TF error and the DLG error plan were 1080 for each sign (positive and negative) of errors, respectively. As examples, the axial, coronal, and sagittal dose difference map for the +30% TF and DLG error in a prostate plan, the +30% TF and DLG error in a head‐and‐neck plan are presented in Figure 1, respectively. All the image‐processing processes presented above were performed with the Image Processing Toolbox of Matlab R2020b (MathWorks).

### 2.2 The CNN modeling for error detection and classification

**FIGURE 2:**
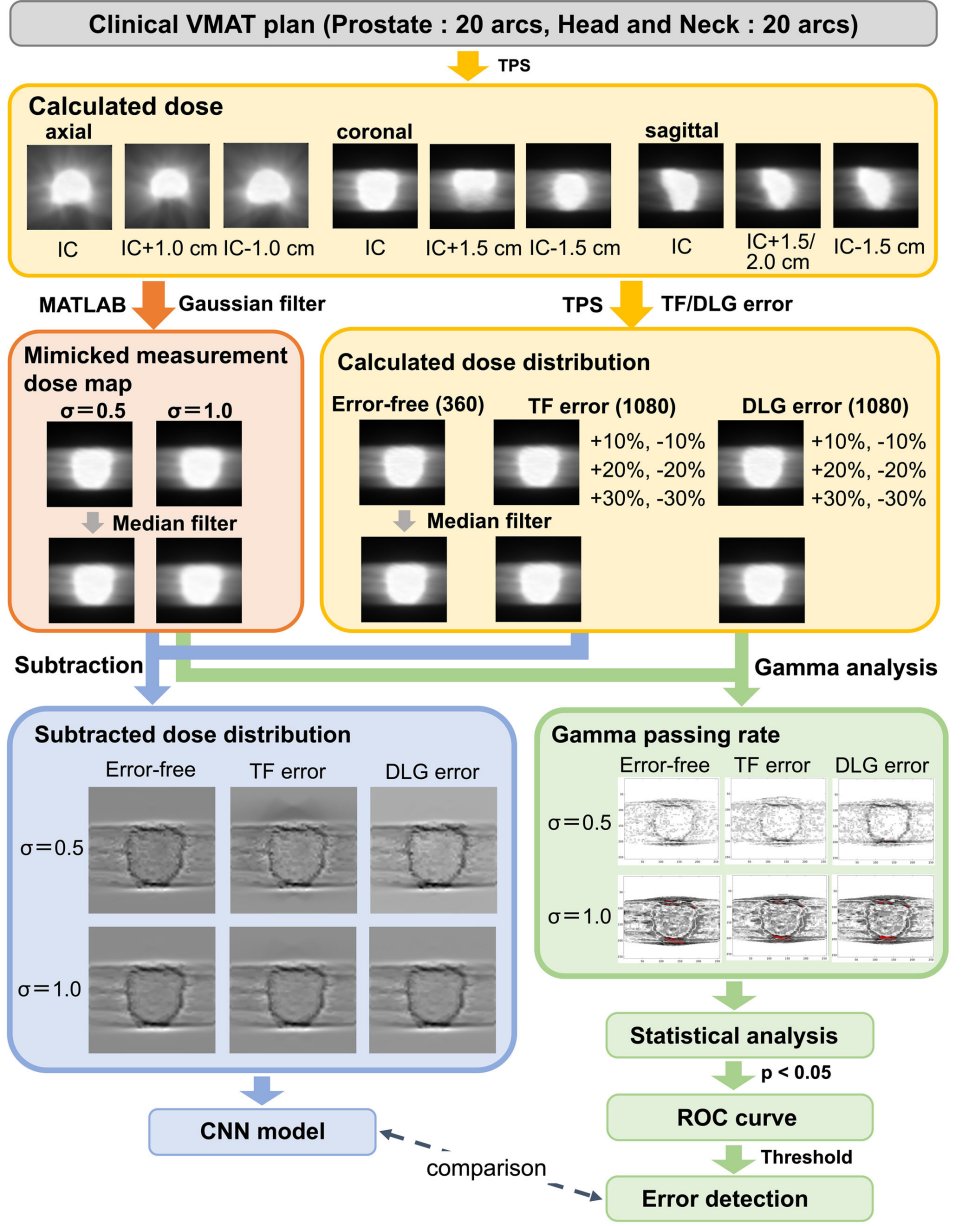
The overall workflow of creating of convolutional neural network (CNN) models and performing the gamma analysis is provided with the number of datasets in parentheses. DLG, dosimetric leaf gap error plans; free, error‐free plans; IC, isocenter; TF, transmission factor error plans.

We constructed CNN models of (1) to detect and distinguish TF error from the error‐free state (“TF error detection model”), (2) to detect and distinguish DLG error from the error‐free state (“DLG error detection model”), and (3) to classify an error as TF error versus DLG error (“2‐class classification model”) using the pre‐trained ResNet‐152 and DenseNet‐201 based on the PyTorch version 2.0.1 implemented on the Google Colab with CUDA version 11.8 and NVIDIA driver version 525.85 (NVIDIA corporation, CA, USA). The overall workflow of constructing of the CNN models is described in Figure 2. The models were trained for 50 epochs using the stochastic gradient descent (SGD) solver with two batch sizes (8 and 16) and two learning rates (0.001 and 0.0001). We performed a data augmentation for the dose difference map of error‐free for training in which a flip, a resizing, a center cropping, and a random erasing were applied. It was ensured that the dose difference map derived from the same clinical plan was not assigned to both the training and test datasets, as one of the pairs (8 arcs) was used as the test dataset, and the remaining (32 arcs) were used as the training dataset. Finally, we assessed quantitatively the performance of the CNN models using the accuracy, sensitivity, specificity, and area under the curves (AUCs).

In order to evaluate the robustness of the created models, we compared them to the other CNN models, namely the ResNet‐101, ResNet‐50, DenseNet‐169, and DenseNet‐161 assuming the same values of the hyperparameters. In addition, the models trained with dataset of dose difference maps of only one plane among axial, coronal, and sagittal were individually created and evaluated. We also created CNN models trained with negative and positive errors grouped separately, namely, (1) the “positive/negative TF error classification model” classifying the positive and negative TF error, and (2) the “positive/negative DLG error classification model” classifying the positive and negative DLG error.

### 2.3 Comparison with the gamma analysis

The performance of the CNN models was compared with the 2D gamma analysis. We calculated the gamma passing rate for the mimicked measured dose map (*σ* = 0.5, 1.0) with error‐containing and error‐free dose distributions. The threshold criteria were set to the global 2%/2 mm and 1%/1 mm, local 2%/2 mm and 1%/1 mm where the criteria of 2% or 1% for dose difference (DD) and 2 or 1 mm for distance‐to‐agreement (DTA) were applied, and regions comprising more than 10% of the maximum dose area were analyzed. The additional criteria of global and local 1%/0.5 mm were set for the DLG error where the DTA is sufficiently smaller than the original DLG value of 0.88 mm. The Wilcoxon signed‐rank test was used to evaluate the difference in the passing rate between error‐free and each error, and *p*‐values less than 0.05 were considered statistically significant. The passing rate of error‐free and each error were considered statistically significant, and we evaluated receiver operating characteristic (ROC) curves and calculated the AUCs to determine a specific threshold of the passing rate for binary classification discriminating between error‐free and each error. We also calculated the accuracy based on the results of classifications.

## 3 RESULTS

### 3.1 The performance of the CNN models for error detection and classification

**FIGURE 3:**
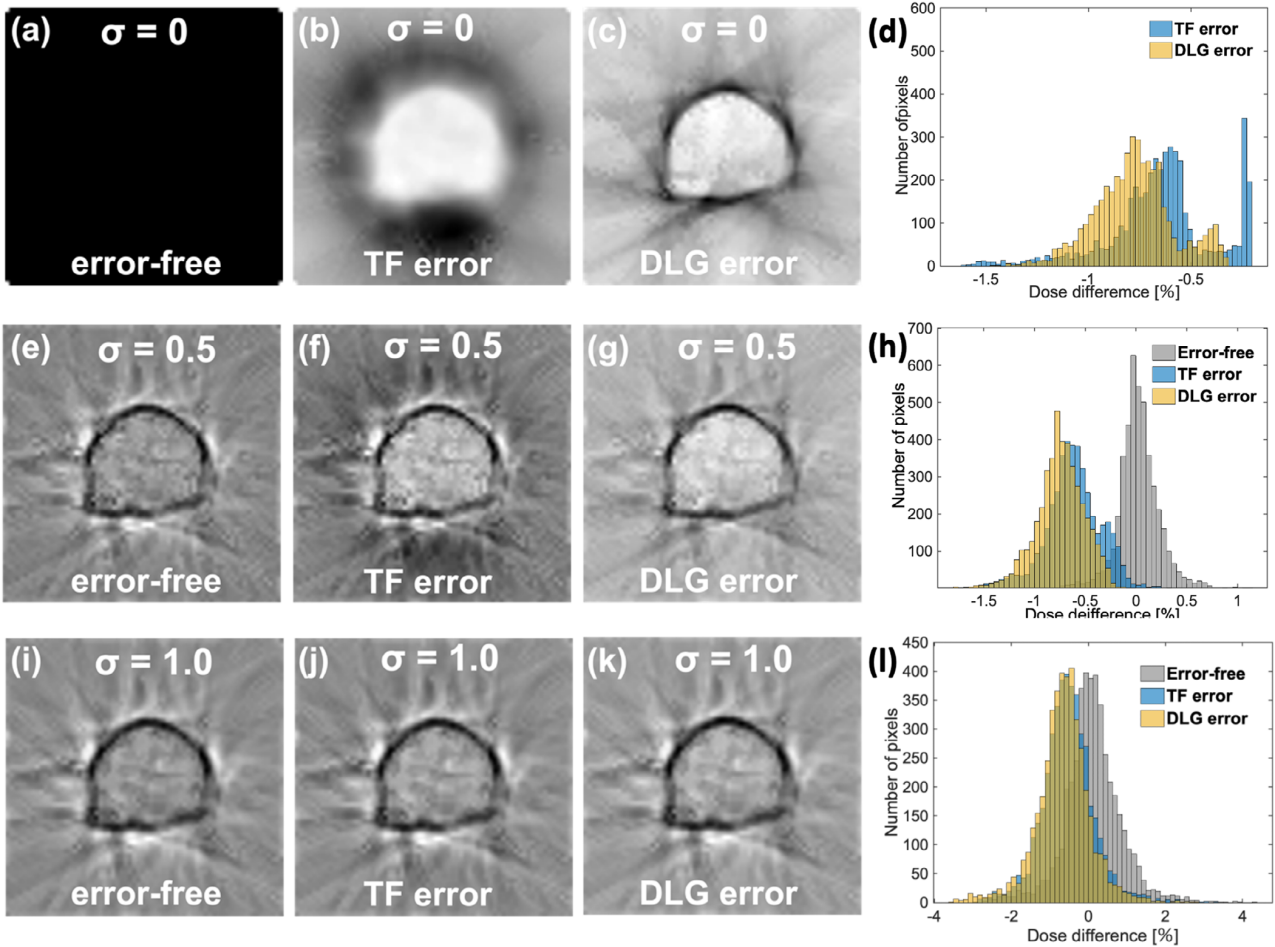
Examples of the dose difference map of error‐free, TF error of +30% and DLG error of +30% and the associated dose difference histogram for a prostate plan created by subtracting the calculated dose distributions from the mimicked measured dose maps for the three degrees of mimicked measurement dose (*σ* = 0, 0.5, 1.0). The figures (a), (b), (c), and (d) are the dose difference maps and the dose difference histograms for *σ* = 0 while (e), (f), (g), and (h) are for *σ* = 0.5, and (i), (j), (k), and (l) are for *σ* = 1.0. DLG, dosimetric leaf gap; TF, transmission factor.

Figure 3 shows the examples of the dose difference map and the associated dose difference histogram (DDH) for a prostate cancer plan with three different mimicked measurement doses. It was observed that the TF and the DLG error exhibit their own specific patterns in the dose difference map for σ = 0, and they become blurred as the value of σ become larger. For a large σ, the DDHs largely overlapped, meaning that it became difficult to discriminate the type of error.

**FIGURE 4:**
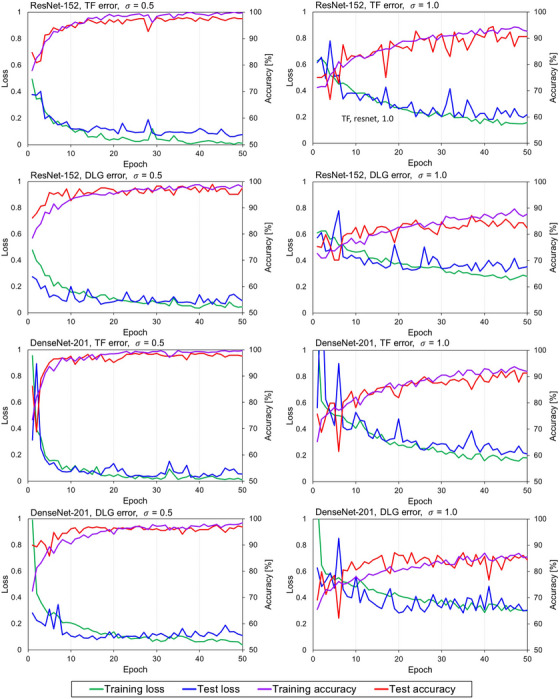
The learning curves (accuracy and loss) for training and test dataset for the TF detection model based on ResNet‐152 (first row) and DenseNet‐201 (third row), and the DLG detection model based on ResNet‐152 (second row) and DenseNet‐201 (fourth row) with *σ* = 0.5 (left‐hand side) and 1.0 (right‐hand side) are shown. DLG, dosimetric leaf gap; TF, transmission factor.

The learning curve for the TF detection model and the DLG detection model with σ = 0.5 and 1.0 are shown in Figure 4. It is observed that the training and test loss were decreased and the training and test accuracy were increased continuously, and those were finally converged at 10−20 epochs for *σ* = 0.5 and 30−40 epochs for *σ* = 1.0, respectively.

Table 1 presents the training and test accuracies for the TF error detection model (“error‐free vs. TF error”) and the DLG error detection model (“error‐free vs. DLG error”) based on ResNet‐152 and DenseNet‐201 for the two degrees of mimicked measurement dose, two batch sizes (8 and 16), and two learning rates (0.001 and 0.0001). Since the batch size of 8 and the learning rate of 0.001 showed the highest accuracy and generalization performance overall, we adopted those values hereafter. In common with all models, the larger the σ of the mimicked measurement dose, the worse the performance of each model became. This can be confirmed by the dose difference maps shown in Figure 2. The detection accuracies of the TF error and DLG error were higher than 95% for *σ* = 0.5 and it was reduced to be around 90% for the TF error detection and in the lower 80% range for the DLG error detection for *σ* = 1.0 as shown in Table 1. The detection performance of the TF error detection model was slightly superior to that of the DLG error detection model: an accuracy of 90.6% for the TF error detection model and an accuracy of 82.3% for the DLG error detection model for *σ* = 1.0 based on ResNet‐152.

**TABLE 1:** The training and test accuracies, sensitivities, specificities, and AUCs of the TF and DLG error detection models based on the ResNet‐152 and the DenseNet‐201 for two degrees of mimicked measurement dose (*σ* = 0.5, 1.0), two batch sizes (8 and 16) and two learning rates (0.001 and 0.0001).

				Accuracy (%)		Sensitivity (%)	Specificity (%)	AUC
	Gaussian filter	Batch size	Learning rate	Training	Test	Test	Test	Test
ResNet‐152								
TF error detection model (error‐free vs. TF error)	*σ* = 0.5	8	10^−3^	99.7	97.6	100.0	90.3	0.998
			10^−4^	98.3	94.4	95.8	90.3	0.974
		16	10^−3^	99.7	96.2	97.7	91.7	0.993
			10^−4^	98.1	95.8	98.1	88.9	0.978
	*σ* = 1.0	8	10^−3^	92.6	90.6	93.5	81.9	0.962
			10^−4^	91.7	85.4	85.2	86.1	0.919
		16	10^−3^	94.6	91.0	92.6	86.1	0.959
			10^−4^	91.5	82.3	86.6	69.4	0.894
DLG error detection model (error‐free vs. DLG error)	*σ* = 0.5	8	10^−3^	98.0	97.6	97.7	97.2	0.994
			10^−4^	96.6	95.8	98.6	87.5	0.989
		16	10^−3^	98.9	95.5	98.6	86.1	0.988
			10^−4^	96.4	94.1	97.7	83.3	0.990
	*σ* = 1.0	8	10^−3^	87.8	82.3	81.9	83.3	0.919
			10^−4^	88.5	83.0	88.4	66.7	0.881
		16	10^−3^	93.1	83.3	91.7	58.3	0.910
			10^−4^	89.6	81.9	88.0	63.9	0.855
DenseNet‐201								
TF error detection model (error‐free vs. TF error)	*σ* = 0.5	8	10^−3^	99.7	97.6	99.5	91.7	0.999
			10^−4^	98.9	95.8	96.8	93.1	0.988
		16	10^−3^	99.5	96.9	98.1	93.1	0.995
			10^−4^	98.1	93.8	94.9	90.3	0.964
	*σ* = 1.0	8	10^−3^	91.9	91.3	93.1	86.1	0.966
			10^−4^	91.8	87.5	91.2	76.4	0.937
		16	10^−3^	94.3	92.4	94.4	86.1	0.963
			10^−4^	92.0	84.7	90.3	68.1	0.905
DLG error detection model (error‐free vs. DLG error)	*σ* = 0.5	8	10^−3^	98.4	96.9	97.2	95.8	0.992
			10^−4^	97.5	96.5	96.8	95.8	0.994
		16	10^−3^	98.5	97.2	97.2	97.2	0.996
			10^−4^	96.7	95.8	98.6	87.5	0.995
	*σ* = 1.0	8	10^−3^	85.5	84.4	86.1	79.2	0.939
			10^−4^	88.9	85.4	90.7	69.4	0.914
		16	10^−3^	89.7	85.1	91.7	65.3	0.920
			10^−4^	89.8	85.1	93.1	61.1	0.899

Abbreviations: AUC, area under the curve; DLG, dosimetric leaf gap; TF, transmission factor.

The results of the ResNet‐152 and the DenseNet‐201 were compared to the other CNN models with the same values of the hyperparameters in Table 2. For the same value of *σ*, the accuracies, sensitivities, specificities, and AUCs were observed fairly stable against the changing the model overall and it was confirmed that the models were robustly created.

**TABLE 2:** The training and test accuracies, sensitivities, specificities, and AUCs of the TF and DLG error detection models based on the ResNet‐152, ResNet‐101, ResNet‐50, DnseNet‐201, DenseNet‐169, and DenseNet‐161 with batch size of 8 and learning rate of 0.001 for two degrees of mimicked measurement dose (*σ* = 0.5, 1.0).

			Accuracy (%)		Sensitivity (%)	Specificity (%)	AUC
	Gaussian filter	Model	Training	Test	Test	Test	Test
TF error detection model (error‐free vs. TF error)	*σ* = 0.5	ResNet‐152	99.7	97.6	100.0	90.3	0.998
		ResNet‐101	99.1	98.3	99.1	95.8	0.998
		ResNet‐50	99.5	96.2	96.3	95.8	0.994
		DenseNet‐201	99.7	97.6	99.5	91.7	0.999
		DenseNet‐169	99.7	96.2	97.2	93.1	0.996
		DenseNet‐161	99.8	97.9	97.7	98.6	0.999
	*σ* = 1.0	ResNet‐152	92.6	90.6	93.5	81.9	0.962
		ResNet‐101	94.0	88.9	87.0	94.4	0.964
		ResNet‐50	93.4	84.4	81.5	93.1	0.949
		DenseNet‐201	91.9	91.3	93.1	86.1	0.966
		DenseNet‐169	94.1	89.6	92.6	80.6	0.945
		DenseNet‐161	94.0	90.3	90.3	90.3	0.969
DLG error detection model (error‐free vs. DLG error)	*σ* = 0.5	ResNet‐152	98.0	97.6	97.7	97.2	0.994
		ResNet‐101	98.8	98.3	98.1	98.6	0.995
		ResNet‐50	97.7	94.8	93.5	98.6	0.992
		DenseNet‐201	98.4	96.9	97.2	95.8	0.992
		DenseNet‐169	98.6	96.2	99.5	86.1	0.994
		DenseNet‐161	99.0	97.6	99.1	93.1	0.997
	*σ* = 1.0	ResNet‐152	87.8	82.3	81.9	83.3	0.919
		ResNet‐101	89.3	83.0	86.1	73.6	0.910
		ResNet‐50	86.0	80.6	81.0	79.2	0.906
		DenseNet‐201	85.5	84.4	86.1	79.2	0.939
		DenseNet‐169	86.2	84.7	93.5	58.3	0.921
		DenseNet‐161	85.9	85.1	91.7	65.3	0.920

Abbreviations: AUC, area under the curve; DLG, dosimetric leaf gap; TF, transmission factor.

Table 3 showed the comparison of the error detection scores for models trained with each of three different planes. Almost the same level of the scores of the TF and DLG error detection were obtained for the three planes with *σ* = 0.5. For the DLG detection with *σ* = 1.0, a notable reduction of the specificity and the AUC were observed for coronal planes, and a superior AUC was obtained for axial planes.

**TABLE 3:** The training and test accuracies, sensitivities, specificities, and AUCs of the TF and DLG error detection models for dose maps in axial, coronal, and sagittal plane based on the ResNet‐152, and the DnseNet‐201, with batch size of 8 and learning rate of 0.001 for two degrees of mimicked measurement dose (*σ* = 0.5, 1.0).

			Accuracy (%)		Sensitivity (%)	Specificity (%)	AUC
	Gaussian filter	Plane	Training	Test	Test	Test	Test
ResNet‐152							
TF error detection model (error‐free vs. TF error)	*σ* = 0.5	Axial	100.0	95.8	97.2	91.7	0.985
		Coronal	98.4	96.9	97.2	95.8	0.990
		Sagittal	100.0	94.8	94.4	95.8	0.989
	*σ* = 1.0	Axial	93.8	88.5	88.9	87.5	0.925
		Coronal	91.1	86.5	95.8	58.3	0.943
		Sagittal	95.1	88.5	93.1	75.0	0.931
DLG error detection model (error‐free vs. DLG error)	*σ* = 0.5	Axial	99.0	97.9	98.6	95.8	0.996
		Coronal	99.0	94.8	97.2	87.5	0.990
		Sagittal	98.7	96.9	97.2	95.8	0.986
	*σ* = 1.0	Axial	88.8	89.6	94.4	75.0	0.960
		Coronal	87.5	72.9	81.9	45.8	0.773
		Sagittal	92.4	79.2	76.4	87.5	0.880
DenseNet‐201							
TF error detection model (error‐free vs. TF error)	*σ* = 0.5	Axial	99.0	95.8	97.2	91.7	0.991
		Coronal	97.7	94.8	97.2	87.5	0.995
		Sagittal	99.0	97.9	97.2	100.0	0.999
	*σ* = 1.0	Axial	91.1	85.4	87.5	79.2	0.947
		Coronal	88.3	86.5	88.9	79.2	0.925
		Sagittal	91.7	78.1	73.6	91.7	0.931
DLG error detection model (error‐free vs. DLG error)	*σ* = 0.5	Axial	98.4	96.9	97.2	95.8	0.991
		Coronal	96.1	95.8	95.8	95.8	0.995
		Sagittal	97.1	91.7	88.9	100.0	0.987
	*σ* = 1.0	Axial	86.2	83.3	81.9	87.5	0.926
		Coronal	82.0	79.2	80.6	75.0	0.873
		Sagittal	84.6	80.2	81.9	86.1	0.883

Abbreviations: AUC, area under the curve; DLG, dosimetric leaf gap; TF, transmission factor.

Table 4 shows the classification scores between the TF and DLG error for *σ* = 0.5 and 1.0. The accuracies, sensitivity, specificity were all larger than 94% and AUC = 0.999 for *σ* = 0.5 and those were reduced to be around 90% and 0.95–0.97 for *σ* = 1.0. respectively.

**TABLE 4:** The training and test accuracies, sensitivities, specificities, and AUCs of the error classification model based on the ResNet‐152 and the DenseNet‐201 for two degrees of mimicked measurement dose (*σ* = 0.5, 1.0), batch size of 8 and learning rate of 0.001.

	Accuracy (%)		Sensitivity (%)	Specificity (%)	AUC
	Training	Test	Test	Test	Test
ResNet‐152					
*σ* = 0.5	99.8	96.8	94.4	99.1	0.999
*σ* = 1.0	97.1	88.0	87.0	88.9	0.948
DenseNet‐201					
*σ* = 0.5	99.2	97.7	98.6	96.8	0.999
*σ* = 1.0	95.5	90.3	90.7	89.8	0.973

Abbreviations: AUC, area under the curve; DLG, dosimetric leaf gap; TF, transmission factor.

**FIGURE 5:**
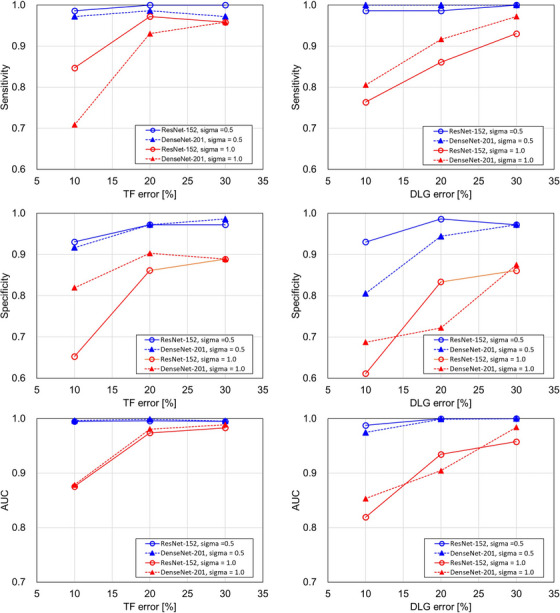
The test sensitivities, specificities, and AUCs of the two CNN models (ResNet‐152 and DenseNet‐201) for the three magnitudes of error (+10, +20, and +30%) and two degrees of mimicked measurement dose (*σ* = 0.5, 1.0). The plot for the TF error detection model (left), the DLG error detection model (upper right) are shown. DLG, dosimetric leaf gap; TF, transmission factor.

Figure 5 shows the sensitivities, specificities, AUCs of the CNN models for the TF error detection model (left) and the DLG error detection model (right) for the three magnitudes of each type of error. The greater the magnitudes of error, the better the performance of the models in detecting the TF and the DLG errors. All the scores were larger than 0.9 overall for *σ* = 0.5. For *σ* = 1.0, the significant reduction of all the indices was observed for the TF and DLG error detection, especially for the specificities and AUCs for DLG error compared to the TF error.

**FIGURE 6:**
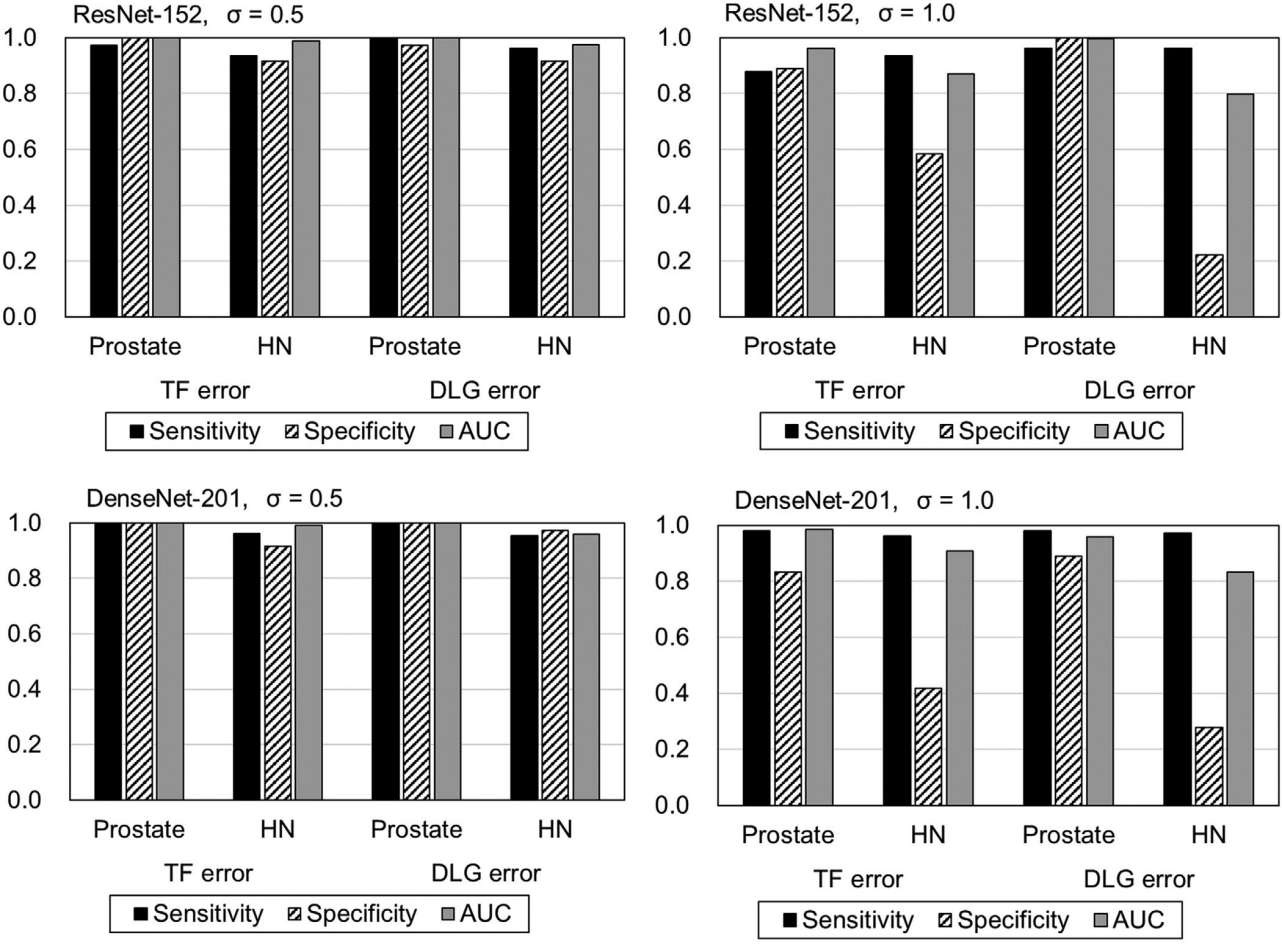
The sensitivities, specificities, and AUCs of the TF and DLG error detection model for two degrees of mimicked measurement dose (*σ* = 0.5, 1.0) for comparison between prostate and head‐and‐neck plans. The upper and lower histograms are the results by the ResNet‐152 and the DenseNet‐201, respectively. DLG, dosimetric leaf gap; EF, error‐free; TF, transmission factor.

Figure 6 shows the sensitivities, specificities, AUCs of each model for each treatment site (prostate and head‐and‐neck) for *σ* = 0.5 and 1.0. The scores of detecting the errors for prostate plans were superior to that of head‐and‐neck plans for all the models. All the scores were larger than 0.9 for *σ* = 0.5 regardless of treatment site. For *σ* = 1.0, all the scores were reduced to be around or less 0.9 except for the DLG error detection of prostate plan. The specificities were notably reduced for head‐and‐neck plans with *σ* = 1.0, especially for the DLG detection.

Table 5 shows the scores for the CNN models in which the sign of the error for each mimicked measurement noise was discriminated. All the models showed fairly high score of classification of the sign of error where at least sensitivity of 93.5%, specificity of 94.0%, and AUC of 0.985 for the DLG error was obtained by ResNet‐152 with *σ* = 1.0.

**TABLE 5:** The training and test accuracies, sensitivities, specificities, and AUCs of the positive/negative classification model for the TF and DLG error based on the ResNet‐152 and the DenseNet‐201 for two degrees of mimicked measurement dose (*σ* = 0.5, 1.0), batch size of 8 and learning rate of 0.001.

		Accuracy (%)		Sensitivity (%)	Specificity (%)	AUC
	Gaussian filter	Training	Test	Test	Test	Test
ResNet‐152						
TF (+) vs. TF (−)	*σ* = 0.5	100.0	100.0	100.0	100.0	1.000
	*σ* = 1.0	99.0	98.4	98.1	98.6	0.999
DLG (+) vs. DLG (−)	*σ* = 0.5	99.8	98.1	100.0	96.3	0.999
	*σ* = 1.0	98.1	93.8	93.5	94.0	0.985
DenseNet‐201						
TF (+) vs. TF (−)	*σ* = 0.5	99.9	99.5	99.5	99.5	1.000
	*σ* = 1.0	98.8	96.8	94.9	98.6	0.998
DLG (+) vs. DLG (−)	*σ* = 0.5	99.7	99.1	100.0	98.1	1.000
	*σ* = 1.0	97.2	94.2	96.3	92.1	0.980

Abbreviations: AUC, area under the curve; DLG, dosimetric leaf gap; TF, transmission factor.

### 3.2 The performance of the gamma analysis for error detection

The values of *p*‐value, AUC, the obtained threshold by the ROC analysis, the accuracy for detecting each error for *σ* = 0.5 and 1.0 for gamma analysis based on the criteria of global 2%/2 mm, 1%/1 mm, 1%/0.5 mm and local 2%/2 mm, 1%/1 mm, 1%/0.5 mm are listed in Table 6. Except DLG error detection with global 2%/2 mm, all the *p*‐values were less than 0.05. All the AUCs were close to or less than 0.5 which that shows a similar performance to the random classifier. The highest accuracy was 74.9% for global 1%/1 mm and *σ* = 1.0. It was concluded that the gamma analysis failed to detect the TF and the DLG error regardless of the degree of mimicked measurement dose map.

**TABLE 6:** The *p*‐value, AUC, appropriate threshold value, accuracy for detecting each error for *σ* = 0.5 and 1.0 for gamma analysis based on the criteria of global 2%/2 mm, 1%/1 mm, 1%/0.5 mm and local 2%/2 mm, 1%/1 mm, 1%/0.5 mm.

	Gaussian filter	*P*‐value	AUC	Threshold (%)	Accuracy (%)
Global gamma analysis, 2%/2 mm					
Error‐free vs. TF	*σ* = 0.5	<0.05	0.495	97.1	26.0
	*σ* = 1.0	<0.05	0.533	98.9	44.5
Error‐free vs. DLG	*σ* = 0.5	0.172	–	–	–
	*σ* = 1.0	0.445	–	–	–
Global gamma analysis, 1%/1 mm					
Error‐free vs. TF	*σ* = 0.5	<0.05	0.476	81.9	25.2
	*σ* = 1.0	<0.05	0.428	98.3	74.9
Error‐free vs. DLG	*σ* = 0.5	<0.05	0.532	99.9	59.4
	*σ* = 1.0	<0.05	0.470	26.7	25.6
Global gamma analysis, 1%/0.5 mm					
Error‐free vs. DLG	*σ* = 0.5	<0.05	0.532	98.5	62.5
	*σ* = 1.0	<0.05	0.459	11.0	25.3
Local gamma analysis, 2%/2 mm					
Error‐free vs. TF	*σ* = 0.5	<0.05	0.495	99.6	55.8
	*σ* = 1.0	<0.05	0.423	58.5	25.2
Error‐free vs. DLG	*σ* = 0.5	<0.05	0.455	85.4	25.1
	*σ* = 1.0	<0.05	0.465	82.2	26.3
Local gamma analysis, 1%/1 mm					
Error‐free vs. TF	*σ* = 0.5	<0.05	0.394	92.6	51.6
	*σ* = 1.0	<0.05	0.394	94.0	74.8
Error‐free vs. DLG	*σ* = 0.5	<0.05	0.472	84.6	30.7
	*σ* = 1.0	<0.05	0.451	12.1	25.3
Local gamma analysis, 1%/0.5 mm					
Error‐free vs. DLG	*σ* = 0.5	<0.05	0.477	86.7	55.1
	*σ* = 1.0	<0.05	0.459	10.1	25.3

Abbreviations: AUC, area under the curve; DLG, dosimetric leaf gap.; TF, transmission factor.

## 4 DISCUSSION

This study consistently has shown that the accuracy of the deep learning‐based models trained with dose difference maps to detect the MLC modeling error depend on the type and magnitude of error, treatment site, and the degree of mimicked measurement dose. The models were considered fairly robust against the selection of batch size and learning rate as shown in Tables 1 and 2, and well‐trained according to the learning curves where training and test accuracy reached a plateau of the similar accuracy as shown in Figure 4.

Sakai et al. showed the results in which the sensitivity of 0.913 for TF error detection model and 0.978 for DLG detection model.^24^ It can be considered comparable to our results with *σ* = 0.5 where the sensitivities of TF and DLG error detection for test dataset were 1.000 and 0.977 as shown in Table 1 for the ResNet‐251, respectively. Overall, the DLG error was found more difficult to detect than the TF error as shown in Table 1 and Figure 5. On the other hand, it is notable that the opposite relationship about the TF and DLG error was found for prostate plans with *σ* = 1.0 for the ResNet‐152 as shown in Figure 6. The VMAT treatment plans for head‐and‐neck cancer are usually more complex than prostate plans and may include the larger area of steep dose gradient as shown in Figure 1. Since the DLG error is pronounced at an area of steep dose gradient, the specific features of the DLG error are easily obscured especially for head‐and‐neck plans by a Gaussian filter applied to dose distribution. For prostate plans, there is a distinctive contour of steep dose gradient along the boundary of PTV even for *σ* = 1.0 as shown in Figure 1. At that area, the DLG error may be particularly pronounced and easily detected. It implies that adjusting the DLG using prostate plans may be preferred, since it is sensitive to the error.

The results presented in Table 3 differentiated three planes (axial, coronal, and sagittal) of dose difference maps in terms of the score of the DLG error detection, whereas the scores of TF error detection were similar for all the planes. The coronal plane was found disadvantageous in detecting DLG error compared to the other planes. It implies that dose gradient may be less prominent in the coronal plane as a general aspect of VMAT plan. In practice, for prostate VMAT plans, there is no organ‐at‐risk on left and right side of target and the degree of intensity modulation on that direction tends to be less.

The results of Tables 4 and 5 show that the created models can accurately discriminate between the TF and DLG errors, and positive and negative errors. The resulted AUC of the classification between the TF and DLG that is 0.948 even for *σ* = 1.0 is superior to that of Sakai et al. in which the AUC of the validation for the TF and DLG classification model was 0.645.^24^ It can be concluded that our deep learning‐based models were superior to extract useful features to distinguish between the TF and DLG error. Indeed, the patterns of positive and negative errors in the dose difference map are quite different and distinguishable. The ability to distinguish the sign of the error is particularly important in optimizing the MLC modeling parameters in TPS commissioning, which is frequently operated near the optimal value corresponding to the error‐free state in our study. Our proposed method can be considered to show proof of the existence of a single deep learning‐based model of specification of the type and positive/negative sign, and estimation of the magnitude of MLC modeling errors. This finding might open up the possibility of an automated optimization of the MLC modeling parameters for VMAT that does not rely on gamma analysis. A possible clinical implementation may be based on a sequential use of multiple models such that a TF error detection and a DLG detection model are applied sequentially, if either one provides “positive” then the corresponding parameter is adjusted, if both provide “positive” then an error classification model is applied in order to specify the dominant type of the error which needs to be eliminated as priority. However, our study also showed that the effect of measurement accuracy is a crucial factor of the feasibility. An EPID may be preferable for its high spatial resolution. Another possibility may be to use multi‐dimensional detector arrays such as Delta4 and the ArcCHECK, however, spatial resolution is possibly a concern especially in adjusting DLG.

As shown in Table 6, regardless of the degree of mimicked measurement dose, it can be concluded that the gamma analysis could not detect the TF or DLG error with all the criteria we adopted namely global 2%/2 mm and 1%/1 mm, and local 2%/2 mm and 1%/1 mm, and the additional 1%/0.5 mm applied for TLG error. The reason why the gamma analysis was extremely inferior in error detection was that the change in the passing rate between the error‐free state and each error was too small meaning that the TF and the DLG error are too small to be detected using gamma analysis. A relatively high accuracy (74.9% and 74.8%) obtained for the TF error detection. However, it is noticeable that the specificity was zero for both cases meaning that the gamma analysis failed to identify error‐free.

Potter et al. investigated an ANN model classifying dosimetric errors including the TF errors and the MU scaling errors using a DDH, and reported that the ANN model provided superior accuracy for detecting and classifying dosimetric errors (96.6% for increased and 94.3% for decreased MLC transmission) to the gamma analysis.^21^ They simulated measurement noise by considering pseudorandom normally distributed noise which can be considered similar to our mimicked measurement dose of σ = 1.0. One of the reasons for the high accuracy of detecting the TF error of their study may be that their simulated TF error was double the original value, corresponding to a “± 100% error” in our study. It is notable that our models showed a high performance forσ = 0.5 for much smaller TF errors, namely, ± 10%, 20%, and 30%; however, it should be noted that σ = 0.5 in our study may correspond to a lower degree of mimicked measurement dose than the one adopted in Potter et al.

There are some limitations in this study. The first one is about the number of dataset. We collected 360 (error‐free) and 1080 (each error) dose distributions and the created models showed a fairly high generalization performance as shown in Figure 4 and Table 1, therefore, we considered the number of our dataset were at a certain level to produce meaningful results. However, since our models were trained only with prostate and head‐and‐neck plans, the models were probably considered body‐site specific, it is then still important to extend our study to the other treatment site and to use dataset of the other institutions in order to improve the generalization performance of the models. How much data must be collected may depend on how many treatment sites need to be considered. Second, we only focused on the MLC modeling error, not other types such as MLC positional errors. One of the associated future tasks is to investigate the case in which an MLC modeling error and positional error coexist. If we create a model to classify all possible patterns and combinations of MLC modeling errors and MLC positional errors, the model would be rather complex and difficult to handle. A realistic procedure might be that we first confirm that MLC positional error is negligible by a MLC dynamic log files analysis for example, and then we start detecting MLC modeling error by applying the proposed model in this study. Third, we simulated measurement doses using a Gaussian filter; no actually measured dose was used. Our proposed method should be tested with measurements performed with 2D or 3D detectors in order to verify the possibility of clinical implementation.

## 5 CONCLUSIONS

We developed deep learning‐based models using dose difference maps to detect and classify MLC modeling errors with a simulated mimicked measurement dose. The models exhibited high detection and classification accuracies for small degrees of mimicked measurement dose and outperformed the conventional gamma analysis. The models were also shown useful in discriminating the sign (positive or negative) of the errors. The results of our study demonstrated that our method can provide an effective and accurate method of MLC modeling in TPS commissioning for VMAT.

## AUTHOR CONTRIBUTIONS

All authors satisfy the authorship requirements and do not have anything to disclose.

## CONFLICT OF INTEREST STATEMENT

The authors declare no conflicts of interest.
